# Multisensory Texture Perception in Individuals with Williams Syndrome

**DOI:** 10.3390/children10091494

**Published:** 2023-08-31

**Authors:** Caroline Cheam, Koviljka Barisnikov, Edouard Gentaz, Fleur Lejeune

**Affiliations:** 1 Department of Psychology, Faculty of Psychology and Educational Sciences, University of Geneva, 1205 Geneva, Switzerland; caroline.cheam@gmail.com (C.C.); koviljka.barisnikov@unige.ch (K.B.); 2 Sensorimotor, Affective and Social Development Unit (SMAS), Faculty of Psychology and Educational Sciences, University of Geneva, 1205 Geneva, Switzerland; edouard.gentaz@unige.ch

**Keywords:** visuo-haptic transfer, intermanual transfer, texture, fabrics, Williams syndrome

## Abstract

The sensory profile of people with Williams syndrome (WS) is characterised by atypical visual and auditory perceptions that affect their daily lives and learning. However, no research has been carried out on the haptic perception, in particular in multisensory (visual and haptic) situations. The aim of this study was to evaluate the communication of texture information from one modality to the other in people with WS. Children and adults with WS were included, as well as typically developing (TD) participants matched on chronological age (TD-CA), and TD children matched on mental age (TD-MA). All participants (N = 69) completed three matching tasks in which they had to compare two fabrics (same or different): visual, haptic and visuo-haptic. When the textures were different, the haptic and visual performances of people with WS were similar to those of TD-MA participants. Moreover, their visuo-haptic performances were lower than those of the two TD groups. These results suggest a delay in the acquisition of multisensory transfer abilities in individuals with WS. A positive link between MA and visual and visuo-haptic abilities only in people with WS suggests that they could benefit from an early intervention to develop their abilities to process and transfer multisensory information.

## 1. Introduction

Williams syndrome (WS) is a rare neurodevelopmental disorder caused by a homozygous deletion of chromosome 7q11.23 [1,2] with an estimated prevalence of 1 in 7500 live births [3]. People with WS have mild to moderate intellectual disabilities [4]. Their cognitive profile is marked by a dissociation between relatively well-developed general language abilities and good performance in visual–perceptual tasks (e.g., object and face recognition) while performance in visual–constructive tasks (e.g., drawing or pattern construction) is severely impaired [4,5,6,7,8,9]. Their sensory profile is characterised by atypical sensory processing in the visual and auditory perception that affects their daily lives and learning [10,11].

Sensory processing corresponds to the way that sensory information (visual, auditory, vestibular, tactile, etc.) is processed at the brain level in order to adopt appropriate behaviours relative to the environment [12]. A recent review identified eighteen studies investigating sensory processing in individuals with WS [10], revealing that only four of them were interested in general sensory processing, while the others focused on hyperacusis. These studies indicated that people with WS presented a high prevalence rate of sensory processing difficulties. John and Mervis [13] administrated the Short Sensory Profile (SSP; [14]) questionnaire to parents of children with WS aged 4 to 10 years and identified two subgroups based on the severity of their sensory difficulties: “high impairment” and “low impairment”. Children in the “high impairment” subgroup had more executive and behavioural difficulties and poorer adaptive functioning. Recently, Powell and Van Herwegen [15] extended these results by showing that the characteristics of these two subgroups varied over time. They identified three distinct developmental trajectories. The first two indicated a stable trajectory between the two assessment times (between 1 and 4 years) whether for the “low” or “high impairment” groups. The third trajectory corresponded to children who had a decrease in sensory processing difficulties over time. These studies provide valuable information on the sensory processing of people with WS but were based on indirect measures. Direct measures of sensory processing could give additional information regarding the sensory difficulties experienced by this population. We will now focus on the visual and haptic modalities that were the subject of this research.

Farran and Wilmut [16] investigated some aspects of visual perceptual processing in WS, and more precisely its segmentation ability regarding texture. In their study, two squares of texture were presented simultaneously and participants were asked to indicate if they were the same or different. Results showed that people with WS had similar comparison performances to those of typically developing (TD) children of the same mental age (6 years), indicating a typical pattern to segment an object into its local parts. In contrast, the integration of local features into a global form is impaired in individuals with WS [17]: they rely on local features when their perceptual grouping is based on alignment, luminance and closure but not on shape, proximity or orientation. This contributes to the deterioration of global perception of the shapes to be reproduced in image production (drawings or construction tasks) and consequently to a degraded overall mental representation. Furthermore, this deficit in perceptual grouping could explain the atypical development of several abilities such as global or local object processing, selective attention and object recognition in people with WS [18].

Nevertheless, object processing could also require other sensory modalities, such as passive or active touch, but only very few studies have assessed tactile perception in people with WS. Recently, Powell and Van Herwegen [15] showed through parental questionnaires that children with WS in the “low impairment” group had considerably greater sensory processing ability than those in the “high impairment” group, especially in touch and oral sensory processing, while still experiencing impairments in all other areas (visual, auditory and vestibular). The authors reported particularities regarding the processing of tactile information in the population with WS, but without proposing a specific explanation. Using direct measures, Yoshioka et al. [19] showed that children and adults with WS aged between 7 and 32 years had difficulties localizing tactile stimuli on their hands. Their error profiles were qualitatively and quantitatively equivalent to those of TD children aged 4 and did not improve with age. The authors suggested that these errors could be linked to the absence of missing genes, which play a role in the development of abilities involving spatial representations, resulting in the poor discrimination of the spatial cues of tactile stimuli. The manual haptic perception of objects (active exploration with the hand) has never been carried out in people with WS.

All activities in our daily lives rely on the interaction and the simultaneous participation of our different senses. For example, the transfer of visual information from one cerebral hemisphere to the other is necessary for object recognition and visuomotor integration [20]. Studies showed that people with WS present atypical interhemispheric communication related to an abnormal anatomical callosal structure [21,22,23,24]. Santos et al. [25] used an image-naming task and showed that individuals with WS and their mental age-matched TD controls exhibited a similar pattern of results: their performances for bilateral presentations were better than for unilateral presentations. Despite this abnormal callosal structure, interhemispheric communication in people with WS remains functional. However, the performance of individuals with WS did not improve with age, suggesting discontinued callosal development. These findings concern the visual modality but no research has been carried out on intermanual transfer (for example, the ability to compare tactile information of a texture between the two hands without visual feedback) in people with WS. As the corpus callosum of people with WS is anatomically abnormal, we can assume that the interhemispheric transfer of tactile information is limited, similarly to that of visual information.

Sensory information processing can also be studied through intermodal transfer. Intermodal transfer is defined as the ability to transfer sensory information from one modality (for example, vision) to another sensory modality (for example, touch). Thus, perceiving an object visually and then recognising it by touch (visuo-haptic transfer), or by hearing (visuo-auditory transfer), allows people to obtain a coherent representation and a better identification of a particular object [26,27]. Studies have shown that individuals with WS integrated visual and auditory information better when the stimuli involved objects or faces whether presented sequentially or simultaneously [28,29,30,31]. In contrast, when the stimuli involved syllables, people with WS failed in the audiovisual transfer [32,33]. To our knowledge, no research has yet focused on visuo-haptic transfer in people with WS, particularly regarding texture information.

The term “texture” corresponds to the physical properties defining the micro-structure of a surface, including elasticity, roughness, hardness, etc. [34,35,36]. Studies on texture have mainly focused on roughness. Movement is essential to perceive texture, as Katz pointed out [37]. This movement occurs whether the subject is rubbing the object with the fingers or the object is moving under immobile fingers. Indeed, as no difference between active and passive tactile manual exploration was found in adults, Heller [38] and Lederman [39] confirmed that texture perception was mainly the result of the processing of cutaneous information rather than kinetics. Srinivasan and La Motte [40] observed similar results with the texture of slightly flexible surfaces like an eraser. In contrast, they observed that adults had better performances in the active condition than in the passive one with very rigid surfaces. Haptic and visual perceptions of texture are equally effective in blindfolded sighted adults and in early-blind and late-blind people but, for the very fine textures of abrasive papers, haptic perception is better than visual perception [38]. In conclusion, the haptic modality seems to be well specialized in the perception of texture [41].

Studies about the visuo-haptic transfer development of everyday texture in the TD population showed that performances increased between 5 and 8 years [42,43]. Using three matching tasks in which participants had to compare two fabrics (same or different), the authors observed that haptic performances were equivalent to visuo-haptic performances, but both were inferior to visual performances. This asymmetry of results could be explained by the perceptual characteristics of the textures: a strong intermodal dissimilarity where texture information perceived in the visual modality is not equivalent to that perceived in the haptic modality [44,45], and a weak bimodal dissimilarity that corresponds to the level of difficulty in discriminating between textures [42]. Another explanation could be related to the sensory experience of participants and their difficulties examining roughness since the texture property has two dimensions, spatial and material [46,47,48]. This may lead to perceptual processing confusion: haptic perception is specialised in the material properties of objects such as roughness, hardness and elasticity [47], in contrast to visual perception, which is the most efficient for assessing spatial properties [35].

Considering the importance of sensory information processing and the lack of studies in the population with WS, the aim of the present study was to evaluate the visual, haptic and visuo-haptic transfer abilities of everyday texture information in people with WS, as well as the link with chronological (CA) and mental ages (MA) on these abilities. In this perspective, we compared their performance to that of TD children and adults with the same CA, and to that of TD children with the same MA, using three simple texture comparison tasks. We expected that

- haptic and visuo-haptic performances would not significantly differ for all participants;
- haptic and visuo-haptic performances would be inferior to visual performances for all participants;
- people with WS would show comparable performance to that of TD children matched on MA for the three tasks;
- people with WS would show inferior performance to that of individuals matched on CA for the three tasks.

## 2. Materials and Methods

### 2.1. Participants

Sixty-nine participants were included in the present study. Twenty-three children (N = 10, age 10–16) and adults (N = 13, age 21–41) with WS aged between 10.1 and 41.7 years old were individually matched to 23 TD children and adults according to their CA (TD-CA), as well as to 23 TD children matched on MA according to their raw score on the Ravens Coloured Progressive Matrices Test (TD-MA) [49]. RCPM is a recognised measure for assessing nonverbal reasoning abilities and considered as fluid intelligence. It is frequently used in studies in WS and largely accepted as a reliable and adequate measure to create control groups matched to people with WS [50]. Table 1 illustrates the participants’ characteristics. All participants with WS received a genetic diagnosis with a positive fluorescent in situ hybridization test.

They were recruited from the Swiss association of Williams–Beuren syndrome and from the French (Rhône-Alpes) association of Williams syndrome. The TD children were recruited through public schools in Switzerland and France, and TD adults were recruited using announcement within the university. Before acceptance, all participants (or their parents) received an information letter and filled out a consent form about the study in accordance with the declaration of Helsinki. The Ethical Committee of the University of Geneva approved the study and the Cantonal Authorities for Primary Education as well as the school administration authorities delivered the authorization. All participants were volunteers and could leave the study at any time.

### 2.2. Stimuli

Eighteen textile fabrics were selected from a set of samples used by Picard [43]: jute, lace, cigarette, tulle, linen, fine cotton, wild silk, jean, thick cotton, fine woollen, lycra, satin, velvet, fine sponge, alcantara, suedette, thick sponge and corduroy. Thick sponge and corduroy were used as two trial items. Each pair of fabric had similar degrees of thickness but they varied according to their tactile roughness. Different types of fabric pairs were made to create two conditions: (1) identical pairs of fabrics with identical fabrics/one fabric and its double, and (2) different pairs of fabrics with similar degrees of tactile roughness and thickness. To limit the influence of colour and brightness in the visual task, all fabrics were made in varying shades of beige. It was controlled that colour/brightness and texture did not co-vary.

### 2.3. Procedure

A trained psychologist individually assessed the participants with WS and TD children in a quiet room at their school and the WS and TD adults in the laboratory at the university. The texture-matching task was administered to all participants, while the RCPM (Ravens test) was only given to participants with WS and to TD-MA participants. Both the RCPM (15 min) and the experimental task (25 min) took approximately 40 min to administer.

**Figure 1:**
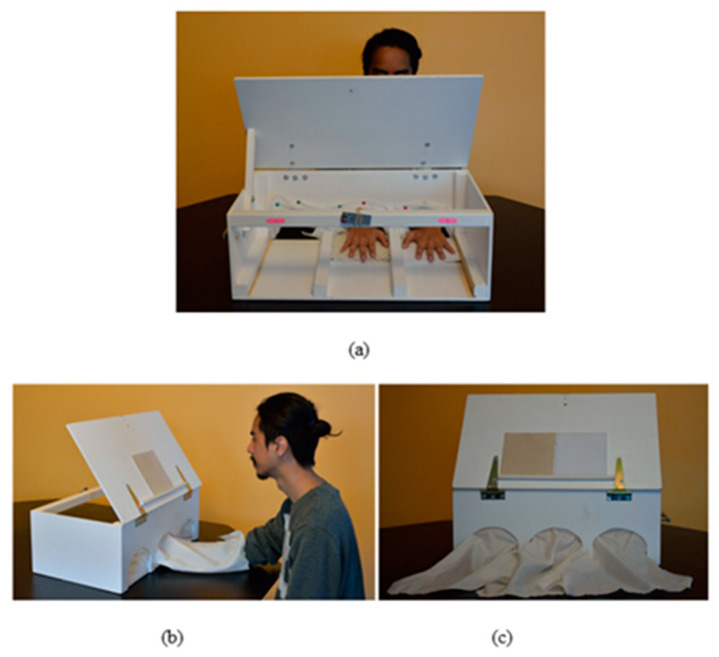
Apparatus of texture-matching tasks: (**a**) haptic task; (**b**) visual-haptic task; (**c**) visual task.

Three matching tasks were given to all participants, systematically presented in the same order: haptic task (H), visuo-haptic task (V-H) and visual task (V). This order of presentation was the same to ensure that participants did not use visual information and experience in the haptic task, which they could have previously learned in the V and V-H tasks. The interval between two tasks was at least 5 min and could be adapted according to the participant’s state of fatigue. Figure 1 illustrates the apparatus of the texture-matching task.

In the haptic (H) task, the participants were asked to haptically compare two fabrics (one per hand) without visual feedback. Participants had to put their two hands in a wooden box with openings hidden by opaque curtains to explore two fabrics without seeing them. Moreover, the experimenter showed them how to evaluate the texture properties with the lateral motion, which is the most effective procedure for processing textures: rubbing their fingers back and forth across the fabrics’ surfaces [51]. This H task assesses the intermanual transfer of texture information.

The visuo-haptic (VH) task aimed to evaluate the intermodal transfer. The participants were asked to compare one fabric, which they tactually explored, with a second fabric, which they visually explored. In the visual modality, the fabrics were presented on an easel with an angle of 60° from the horizontal axis. In the haptic modality, we asked the participants to explore the fabric with one hand: 50% of trials with the right hand and 50% with the left hand. The haptic and the visual stimuli were presented simultaneously.

In the visual (V) task, the participants were asked to visually compare two fabrics presented on the easel.

For each task, two training trials were administrated. Then, the participants had to compare 24 fabric pairs (=24 trials) presented in random order. For each trial, the participants were asked to simultaneously compare two fabrics and to give an oral answer: “same” or “different”. The interval between two trials was between 5 and 10 s. As soon as the participants gave their answer, the next trial began. For each task, 66% of the presented pairs of fabrics were identical (16/24) and the other 33% (8/24) were different. The identical pairs correspond to each fabric used in the different pairs. The fabric presentation duration was not limited but participants’ answers never exceeded 1 min. The percentage of correct answers for each task was calculated.

### 2.4. Statistical Analyses

A data analysis was conducted using SPSS 22.0 (IBM SPSS Statistics, IBM Corporation). The performances were not normally distributed and hence, non-parametric tests were performed. For each type of fabric pair (identical pairs and different pairs), the performances of participants with WS were compared to those of TD-CA participants and to those of TD-MA participants using Mann–Whitney comparisons for each task (H, VH and V). Subsequently, Friedman ANOVAs were performed to compare the three tasks (H, VH and V) for each group (WS, TD-CA and TD-MA). For these analyses, the significance level was 0.05. The effect size for nonparametric tests was reported (r-values). Finally, for each type of fabric pair (identical pairs and different pairs), Spearman correlations were conducted to investigate the link between the matching performances and, respectively, the chronological age and the raw scores of RPCM. For these correlations, the significance values were adjusted with a Bonferroni correction for these multiple tests (12). Thus, the significant threshold was 0.004 (0.05/12).

## 3. Results

### 3.1. Identical Pairs

Table 2 presents the descriptive data of the participants with WS and TD-CA and TD-MA participants regarding correct identification for identical pairs of fabrics in the three tasks.

#### 3.1.1. WS Group versus TD-CA Group

Mann–Whitney comparisons for each task indicated that TD-CA participants and those with WS showed similar performances in the V (*p* = 0.99), H (*p* = 0.50) and VH tasks (*p* = 0.46). Friedman ANOVAs revealed a significant effect of the task in the TD-CA group (χ^2^ = 19.8, *p* < 0.001), but not in the group with WS (*p* > 0.05). For the TD-CA group, the Wilcoxon pairwise comparisons revealed higher performances in the V task (M = 95.4%, SD = 5.1) than in the H (M = 84.5%, SD = 13.3) (T = −1.022, *p* = 0.001, r = −0.51) and VH tasks (M = 84.2%, SD = 12.5) (T = −1, *p* = 0.001, r = −0.49). However, the performances in the VH and H tasks did not differ significantly (*p* = 1).

#### 3.1.2. WS Group versus TD-MA Group

Mann–Whitney comparisons for each task showed that TD-MA participants and those with WS showed similar performances in the V (*p* = 0.93), H (*p* = 0.12) and VH tasks (*p* = 0.73). A Friedman ANOVA showed a main effect of the task in the TD-MA group (χ^2^ = 10.4, *p* = 0.006), but not in the group with WS (*p* > 0.05). The Wilcoxon pairwise comparisons revealed that the TD-MA participants had significantly lower performances in the H task (M = 87.8%, SD = 10.1) than in the V (M = 93.8%, SD = 6.5) (T = −0.74, *p* = 0.012, r = −0.37) and VH tasks (M = 93.2%, SD = 8) (T = −0.69, *p* = 0.018, r = 0.35). Finally, their V and VH performances did not differ significantly (*p* = 1).

#### 3.1.3. Correlations between Matching Performances and Respective Chronological Age and RCPM Scores

The results did not show any significant correlation between chronological age and all matching performances for either TD participants or those with WS (every *p* > 0.004). Similarly, the results did not show any significant correlation between RCPM scores and all matching performances for TD-MA participants and those with WS (every *p* > 0.004).

### 3.2. Different Pairs

Table 3 presents the descriptive data of the participants with WS and TD-CA and TD-MA participants regarding correct identification for different pairs of fabrics in the three tasks.

#### 3.2.1. WS Group versus TD-CA Group

Mann–Whitney comparisons for each task revealed that the TD-CA group had significantly better scores in the H (z = −3.38, *p* = 0.001, r = −0.49) and VH (z = −4.96, *p* < 0.001, r = −0.73) tasks than the group with WS. This group difference is not significant in the V task (z = −1.84, *p* = 0.066, r = −0.27).

The Friedman ANOVA showed a main effect of the task in the TD-CA group (χ^2^ = 27.1, *p* < 0.001) and in the group with WS (χ^2^ = 27.4, *p* < 0.001). The Wilcoxon pairwise comparisons showed that the TD-CA participants performed significantly better in the V task (M = 91.8%, SD = 8.9) than in the H (M = 66.3%, SD = 13.3) (T = 1.26, *p* < 0.001, r = 0.63) and VH tasks (M = 68.5%, SD = 16.8) (T = 1.22, *p* < 0.001, r = 0.61). However, no significant difference between the performances in the VH and H tasks was found (*p* = 1). Similar results were observed for the participants with WS. They had significantly better performances in the V task (M = 76.6%, SD = 29) than in the H (M = 38%, SD = 27.6) (T = 0.98, *p* = 0.001, r = 0.49) and VH tasks (M = 26.6%, SD = 22.4) (T = 1.44, *p* < 0.001, r = 0.72). Their performances did not differ significantly between the VH and H tasks either (*p* = 0.37).

#### 3.2.2. WS Group versus TD-MA Group

Mann–Whitney comparisons for each task indicated that the TD-MA participants and those with WS showed similar performances in the H (*p* = 0.19) and V tasks (*p* = 0.31). However, in the VH task, participants with WS had significantly lower performances (M = 26.6 %; SD = 22.4) than the TD-MA participants (M = 43.5%; SD = 25.5) (z = −2.22, *p* = 0.03, r = −0.33).

The Friedman ANOVA showed a significant effect of the task in the TD-MA group, (χ^2^ = 28, *p* < 0.001) as well as in the group with WS (χ^2^ = 27.4, *p* < 0.001). The Wilcoxon pairwise comparisons revealed that the TD-MA participants had significantly higher performances in the V task (M = 85.9%, SD = 19.7) than in the H (M = 47.8%, SD = 25.5) (T = −1.30, *p* < 0.001, r = −0.65) and VH tasks (M = 43.5%, SD = 25.5) (T = 1.30, *p* < 0.001, r = −0.65). However, the performances in the VH and H tasks did not differ significantly (*p* = 0.37). The participants with WS obtained similar results (previously described in the section concerning the comparison with TD-CA participants).

#### 3.2.3. Correlations between Matching Performances and Respective Chronological Age and RCPM Scores

No significant correlation was found between chronological age and all matching performances for either TD participants or those with WS (every *p* > 0.004). Moreover, the results did not show any significant correlations between RCPM scores and matching performances for the TD-MA participants in all exploration tasks. However, concerning the participants with WS, these correlations were positive and significant in the VH (rs = 0.83, *p* < 0.001) and V tasks (r = 0.59, *p* = 0.003) but not in the H task.

## 4. Discussion

The aim of this study was to investigate the ability to simultaneously process and transfer tactile and visual texture information in children and adults with WS. To do this, we compared their performance to that of TD children and adults with the same CA (TD-CA), and to that of TD children with the same MA (TD-MA). Results indicated that children and adults with WS were able to identify identical pairs of fabrics (around 90%) very well and their performances were similar in the three perceptual tasks. In contrast, the performances of TD participants differed according to the task: a superiority of visual processing over other sensory processing was revealed, which is in line with previous studies [26,42,43]. Moreover, the TD-CA group had a different pattern of results in comparison to the TD-MA group: TD-MA participants performed better in the visuo-haptic task than in the haptic task, and it was similar to the visual task. In contrast, TD-CA had lower performances in the visuo-haptic task than in the visual task, and it was similar to the haptic task. It is possible that lower-level processing was used by TD-MA participants but was efficient enough to identify two similar textures in the visuo-haptic task. According to Gori et al. [52], the physical maturation of the body would play a role in the calibration of the sensory systems and the visuo-haptic integration development is incomplete before 8 years of age. This calibration of the sensory systems during development would induce a finer sensory processing that could lead TD-CA participants to wrongly identify differences between two similar textures.

Secondly, for all participants, identifying different pairs was more difficult (60.6%). Indeed, when two textures are different, a more elaborate analysis is necessary: a detailed analysis of all the features of the texture, followed by a comparison of this new global representation with the previous one being carried out [53,54]. Moreover, a similar pattern was present in our three populations: participants had lower performances in the haptic and visuo-haptic tasks than in the visual task, like those obtained by TD children and adults in Picard’s studies [42,43]. During the visuo-haptic task, TD-CA participants could have some difficulties using and comparing the relevant texture information, with some conflict between roughness and relief information. Indeed, vision essentially helped participants process the spatial properties of textures, typically their relief surface, to compare the two fabrics. In contrast, touch helped them process the basic properties of tactile textures (softness and thickness) rather than their relief properties [46,47,48]. The perceptual equivalence was only partial, as indicated by differences between the visual and tactile performances. Consequently, if perceptual equivalence was not attained, the visuo-haptic transfer of texture information was limited in the three groups.

The visual performance of participants with WS in discriminating two different textures was lower than that of TD-CA participants but equivalent to that of TD-MA participants, in other words, similar to that of TD children with a mean age of 6.3 years (range from 5 to 8.9 years). These results are consistent with those of Farran and Wilmut [16]: individuals with WS had similar visual texture discrimination compared to TD individuals of the same MA. Taken together, these results could be explained by difficulties of individuals with WS in perceptual grouping, leading to a degradation of the overall representation. To further assess this hypothesis, it would be interesting to replicate the tasks of Farran and Wilmut [16] and to determine if a correlation exists between the scores of their visual texture comparison and those of our texture-matching task.

The performance of participants with WS in the haptic task in discriminating two different textures was similar to that of TD-MA children, but lower than that of TD-CA participants. Participants with WS would present the same difficulties in selective attention as young children, and more precisely difficulties in tactually selecting the relevant information necessary to identify differences between two textures [26]. It is possible that the large amount of information to process simultaneously could confuse people with WS during the haptic comparison of fabrics. These results are in accordance with those of Yoshioka [19]: haptic performances of people with WS are equivalent to those of TD people of the same MA matched on their level of analogical reasoning, in other words, to those of TD children aged 6.3 years. The difficulties in perceptual localization that people with WS present with their hands seem to have repercussions on their haptic processing of textures. Furthermore, this haptic processing during a simultaneous presentation requires an intermanual transfer of tactile information. We can therefore assume a delay in the acquisition of intermanual transfer abilities in people with WS, probably related to their atypical interhemispheric communication related to an abnormal anatomical callosal structure [21,22,23,24].

The visuo-haptic performance of people with WS was lower than that of the two TD control groups when they had to discriminate two different textures. We did not expect such a result but rather assumed comparable performance between participants with WS and TD-MA children with the same MA. The visuo-haptic transfer of texture information therefore seems particularly difficult for children and adults with WS, as also reported by Böhning et al. [32] and D’Souza et al. [33] with other sensory modalities. These authors observed that people with WS had difficulty integrating auditory and visual information during an audiovisual syllable transfer task. In their view, this population would not be able to integrate multisensory information. Other studies have shown that people with WS had atypical sensory processing that was related to elevated levels of severe anxiety symptoms [55] and repetitive interests or routine behaviours [56]. Wuang and Tsai [57] found that multisensory processing difficulties in 6- to 12-year-old children with WS were associated with lower participation in school activities and poor adaptive behaviours. Our results reinforce these conclusions by demonstrating that these multisensory difficulties encountered by individuals with WS are also observed during visual–haptic texture transfer.

The link between chronological and matching performances was analysed and no significant correlation was found. It seems that chronological age is not a good predictor of texture comparison performance. Picard [43] observed an increase with age for such skills between 5 and 8 years. It should be noted that the youngest participants with WS were 10 years old, as well as their chronologically age-matched peers. At this age, previous research showed that haptic exploration was similar to that of TD adults [52]. Consequently, the intra- and intermodal performances of our participants were likely to be at their optimal level. It is therefore possible that we observed a significant impact of chronological age in a younger population of children with WS. Indeed, a recent study showed a developmental progression in verbal and non-verbal abilities in WS, especially among younger children [58]. However, no significant correlation was found in TD-MA participants who had a chronological age ranging between 5.0 and 8.9 years. The age range may not be large enough to reveal a significant effect of age in this group. Further investigations are needed to evaluate the possible effect of chronological age on the abilities to process and transfer, simultaneously, tactile and visual texture information in young children with WS.

Interestingly, when the pairs were different, our results showed two significant and positive correlations between visual and visuo-haptic comparison scores and the nonverbal reasoning score in participants with WS only. Firstly, individuals with lower scores on nonverbal reasoning ability, assessed with the RCPM test, appear to be the ones who had more difficulty visually identifying two different textures. Similarly, Wuang and Tsai [57] found that the cognitive level (IQ) measured with the WISC-III was strongly related to visual discrimination performance in children with WS. Secondly, the level of nonverbal reasoning ability also seems to have strong impact on the visuo-haptic texture comparison. Indeed, our participants with WS who had higher scores on the RCPM test were better at identifying two different textures, implying a more elaborate sensory processing [47]. Rose et al. [59,60] showed that the performance in a visuo-haptic transfer task in preterm and full-term infants was predictive of their later cognitive abilities during childhood, albeit the effect was weak. Moreover, the work of Giannopulu, Cusin, Escolano and Dellatolas [61] showed that bimanual visuo-haptic recognition performance correlated significantly with linguistic cognitive abilities and with visuo-constructive abilities. Thus, the authors concluded that the evaluation of visuo-haptic recognition would be a relevant tool for the early identification of children at risk of learning difficulties, as well as for children with intellectual disabilities, motor deficits or other neurodevelopmental disorders. Our results reveal a positive link between nonverbal fluid intelligence and visual and visuo-haptic abilities of people with WS, confirming the importance of considering mental age when assessing these abilities.

Limitations on the generalizability of our findings should be acknowledged. It should be noted that we only used one fabric pairing level corresponding to the most difficult level (each fabric in a pair shared the same or similar degrees of roughness and thickness), resulting in a very low success rate in people with WS. Picard [43] used several fabric pairing levels in a visuo-haptic texture transfer task with 5- and 8-year-old TD children: they obtained visuo-haptic performance close to the ceiling effect when the fabric pairing level was easy (different roughness and different thickness) or intermediate (same roughness and different thickness, or different roughness and same thickness). However, when the fabric pairing level was difficult, their performance was lower but not below the chance. Future studies would be interesting to examine the effect of different degrees of the fabric pairing level in people with WS.

## 5. Conclusions

Our study provides new insights for a better understanding of sensory processing in children and adults with WS, more precisely, the ability to simultaneously process and transfer tactile and visual texture information. It highlights a delay in the acquisition of interhemispheric and intermanual exchange abilities in individuals with WS, but also their sensory integration difficulties, which can disrupt their daily functioning and learning abilities. Moreover, this research reveals a positive link between mental age and visual and visuo-haptic abilities only in people with WS, suggesting that individuals with WS could benefit from an early intervention to develop their abilities to process and transfer visuo-haptic information and in turn improve their daily activities and learning.

## Figures and Tables

**Table 1:** Means (and standard deviations) of chronological ages, Ravens Coloured Progressive Matrices scores and percentages of females and right-handed participants according to the group (WS, TD-CA, TD-MA).

	WS N = 23	TD-CA N = 23	TD-MA N = 23
Chronological Age; Mean (SD)	21.9 (9.7)	21.8 (9.8)	6.3 (1.2)
% Females	43.5	47.8	47.8
% Right-handed	87	91	91
RPCM Score; Mean (SD)	17.7 ^(a)^ (7.9)	_ ^(b)^	18 ^(c)^ (7.6)

Notes: ^(a)^ According to the RPCM’s norms [49], this score corresponds to a TD child’s, aged between 6.8 and 7.2 years, level of logical reasoning at the 25th percentile; ^(b)^ the RPCM was not given to this group because this test was designed only for children aged between 5.0 and 11.11; ^(c)^ the chronological age of this group was comprised between 5 and 8.9 years.

**Table 2:** The percentage of correct answers (means and standard deviations) for the identical pairs in each task (haptic, visual and visuo-haptic) and in each group of participants (WS, TD-CA and TD-MA).

Tasks	TD-CA	TD-MA	WS
Visual	95.4 (5.1)	93.8 (6.5)	92.7 (10.6)
Haptic	84.5 (13.3)	87.8 (10.1)	87.5 (11.9)
Visuo-haptic	84.2 (12.5)	93.2 (8)	86.7 (13.5)

**Table 3:** The percentage of correct answers (means and standard deviations) for the different pairs in each task (haptic, visual and visuo-haptic) and in each group of participants (WS, TD-CA and TD-MA).

Tasks	TD-CA	TD-MA	WS
Visual	91.8 (8.9)	85.9 (19.7)	76.6 (29)
Haptic	66.3 (13.3)	47.8 (25.5)	38 (27.6)
Visuo-haptic	68.5 (16.8)	43.5 (25.5)	26.6 (22.4)

## Data Availability

Data supporting the findings of this study are available from the corresponding author on a reasonable request.
